# Pectoralis major rupture in body builders: a case series including anabolic steroid use

**DOI:** 10.1186/s12891-023-06382-1

**Published:** 2023-04-04

**Authors:** Nikolaos Stefanou, Nikolaos Karamanis, Effrosyni Bompou, Dionysia Vasdeki, Thomas Mellos, Zoe H. Dailiana

**Affiliations:** 1 Department of Orthopaedic Surgery, Faculty of Medicine, School of Health Sciences, University of Thessaly, 3 Panepistimiou St, 41500 Biopolis, Larissa, Greece; 2 Department of Hand, Upper Extremity Surgery and Microsurgery, IASO Thessalias, 41500 Larissa, Greece; 3 Department of General Surgery, Faculty of Medicine, School of Health Sciences, University of Thessaly, 3 Panepistimiou St, 41500 Biopolis, Larissa, Greece; 4 B’ Department of Orthopaedic Surgery-Sports Medicine, IASO Thessalias, 41500 Larissa, Greece

**Keywords:** Pectoralis major muscle/tendon, Anabolic steroids, Weightlifting, Bodybuilding, Case report

## Abstract

**Background:**

Rupture of the pectoralis major (PM) muscle is a rare injury, with increasing incidence over the last decades, mainly due to participation in weightlifting and contact sports. Surgical management of PM injuries has been related with superior functional outcome, faster return to activities, better cosmesis and higher level of patients’ satisfaction. The aim of the study is to present our experience in the management of this rare clinical entity and to correlate the use of anabolic steroids in the occurrence of the injury and the impact of type of injury, method of reconstruction and anabolic steroids on the post-operative outcome*.*

**Cases:**

We present a series of six male bodybuilding athletes who sustained PM rupture during weightlifting. We recorded the location & type of injury, the history and type of anabolic steroids use, the method of repair and the post-operative outcome.

**Treatment and outcomes:**

The mean follow-up period was 16 (12–24) months. All patients treated surgically had excellent results according to Bak criteria and returned to full activity within 5.4 (5–7) months following surgical reconstruction. No post-operative complications were recorded, despite the continued use of anabolic steroids, however one patient died from myocardial infarction within a year of surgical treatment.

**Conclusions:**

PM rupture is an injury with increasing incidence within bodybuilding athletes probably not related to the dominance of the limb. Fixation of the tendon with suture anchors results in excellent clinical outcome and patient’s satisfaction postoperatively regardless the chronicity of the repair. Our observations in these cases suggest that anabolic steroids use may contribute to the injury due to an excessive upward adjustment of the athlete's goals in lifting weights and moreover the continuation of administration even in the recovery period does not seem to have a negative effect either on the time or on the level of adequate functional recovery postoperatively.

## Background

Rupture of the pectoralis major (PM) muscle is a rare injury, which was first described by Patisser in 1822 [1, 2]. These injuries most commonly occur in active men aged 20 to 40 years as they approached the lowest point of the bench press repetition, where pectoralis major tendon is most prone to rupture during eccentric contraction with the arm positioned in 30 degrees of extension and 40 degrees of abduction [3–5]. Over the last decades the overall incidence in the general population has increased, more likely due to the increased participation in competitive-contact sports and weightlifting training in combination with the use of anabolic steroids, which probably result in stiffer tendons with less elongation due to collagen dysplasia, increased vascularization and cellularity, and microdamage of collagen fibers [4, 6].

In 1980 Tietjen divided the injuries into the following types: type I, sprains, or contusion; type II, partial tears of the muscle; and type III, complete tears. The injuries were further divided by the location of the tear; sternoclavicular (A), muscle belly (B), musculotendinous junction (C) and insertion to the bone (D) [7]. El Maraghy et al. proposed a different classification system, considering the bilaminar morphology of the tendon [5]. He divided ruptures based on the extend of injury’s width, the extend of injury’s thickness, the timing of injury and its location. Ruptures were divided in complete or incomplete (width), full or partial (thickness), acute or chronic (timing) and located at: the origin of the muscle/muscle belly, at/within the musculotendinous junction, or at the tendinous insertion with or without bony avulsion [5, 8]. Most commonly the ruptures are located at the level of humeral insertion, followed by ruptures at the musculotendinous junction. The speed of the force that produces the rupture can influence the rupture site. Low speed forces, as the ones produced during training and wrestling, result in ruptures at the insertion site, while high speed forces, mainly related to work related injuries, result in ruptures at the musculotendinous junction [3].

Nonoperative management is traditionally indicated and well tolerated in patients with intramuscular injuries, strains, and partial tears or in low-demand patients. Surgical management of PM rupture is highly recommended for complete tears and for high demand patients who wish to achieve functional recovery of arm adduction, forward flexion, and internal-rotation strength [8]. Operative management has been associated with superior functional outcome and return to previous sport activity levels. Additionally, it restores cosmesis and provides higher levels of patient’s satisfaction. Several surgical techniques have been described in the literature, especially regarding the fixation method of the muscle’s tendon to the humerus [9–11].

The aim of this study is to present our experience in the management of six bodybuilding athletes who sustained rupture of the pectoralis major muscle during weightlifting activities. We recorded the side and the type of PM injury, the use of anabolic steroids and the method of reconstruction, aiming to correlate the use of anabolic steroids in the occurrence of the injury and the impact of type of injury, method of reconstruction and use of anabolic steroids on the post-operative outcome.

## Cases

We retrospectively studied six consecutive patients, bodybuilding athletes who sustained PM rupture and treated by 2 groups of surgeons in the same city during the same time period (September 2017-September 2020). Informed consent for participation to the study was obtained from every patient. All patients were male with a mean age of 29.17 years (21–40 years). The injury involved the left, non-dominant, arm in 3/6 patients and the dominant right hand in other 3/6 patients. Patients’ history did not include any relevant past injuries or therapeutic interventions.

The mechanism of injury, in all cases, included excessive muscle tension, while performing flat bench press weightlifting exercises during which the arm is abducted and externally rotated, with the pectoralis major tendon being under maximum tension. Sometimes, due to muscle fatigue or weakness, the weight slips to one side leading to eccentric contraction of the pectoralis major and subsequently to rupture.

**Fig. 1:**
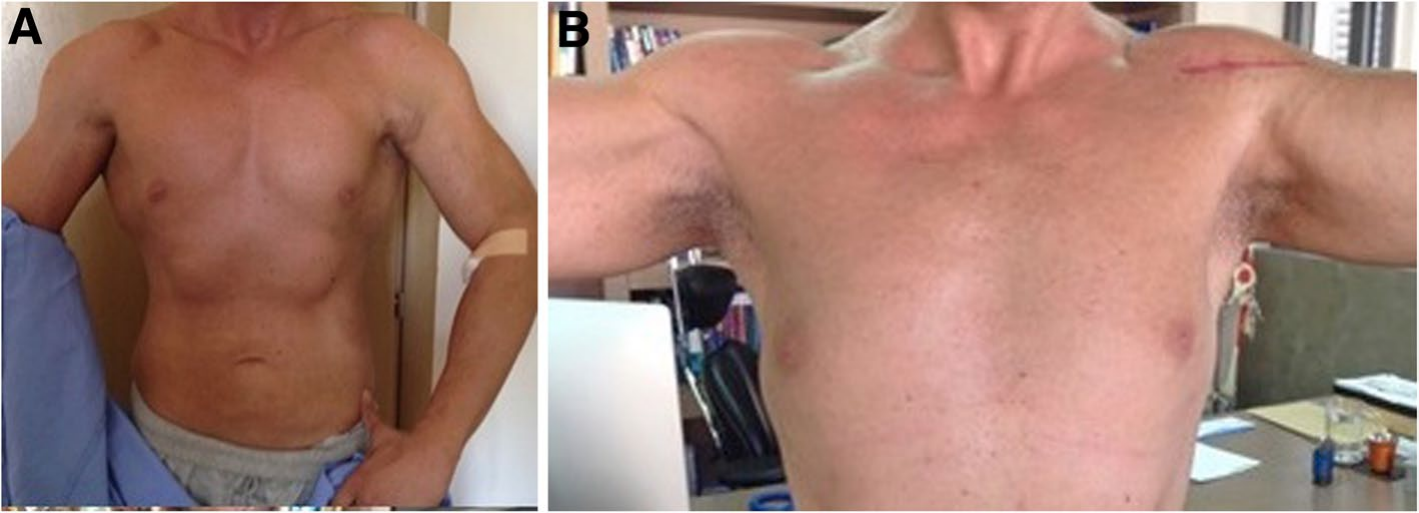
**A.** A 29-years old patient who sustained a complete rupture of the left PM at the site of insertion onto the humerus. Deformity of the anterior axillary fold and weakness with abduction and internal rotation of the arm were evident at the time of presentation. **B** Excellent restoration of the anterior axillary fold and functional outcome 3 months postoperatively

In the acute setting, the symptoms included pain and inability to continue training, while the clinical signs included cosmetic deformity of the anterior axillary fold accompanied by the characteristic "dropped nipple" sign, ecchymosis overlying the chest wall and proximal biceps muscle, and a side-to-side asymmetry with resisted horizontal adduction and internal rotation (Fig. 1a). Radiographs were performed in all cases to exclude the rare bony avulsion type IIIE of Bak’s classification and Magnetic Resonance Imaging (MRI) were performed in all patients at presentation, to classify the injury.

Three of six patients confirmed the use of anabolic steroids during the time of PM rupture (bulking cycle) and reported use for at least one year period. Two of them used dianabol 15 mg daily, for 8 weeks and one trenbolone enathanate 150-300 mg, injected per week, for 8 weeks. Testosterone (propionate/ethanate blend) 250 mg, injected per week, for 8–10 weeks was used simultaneously in some of the bulking cycles. Ιn the interim they all used Arimidex in the dosage of 1 mg per day, for 4 weeks and Liv 52 in the dosage of 1–2 caps, 2 times per day for 4 weeks.

PM injuries were classified according to the Tietjen classification system [5]. Five patients (83.3%) sustained complete rupture of the tendon at the site of insertion onto the humerus (type IIID) and one patient (16.7%) had a complete PM rupture at the musculotendinous junction site (type IIIC).

PM repair was classified as acute, if the injury was sustained in less than six weeks from the time of presentation and chronic, if that was older than 6 weeks [2, 8]. Based on this, in our series five patients were classified as acute and the remaining one was regarded as chronic, treated 30 months after injury (Table 1).

**Table 1:** Patients’ demographic and injury characteristics and use of steroids

	Gender	Age	Location of injury	Type of injury	Chronicity of injury	Management of injury	Use of steroids
1	Male	23	Tendon insertion into humerus	Complete	Chronic	Fixation with suture anchors	Yes
2	Male	29	Tendon insertion into humerus	Complete	Acute	Fixation with suture anchors	Yes
3	Male	30	Musculotendinous junction	Complete	Acute	End-to-end sutures within musculotendinous junction	Yes
4	Male	21	Tendon insertion into humerus	Complete	Acute	Fixation with suture anchors	No
5	Male	40	Tendon insertion into humerus	Complete	Acute	Fixation with suture anchors	No
6	Male	32	Tendon insertion into humerus	Complete	Acute	Fixation with suture anchors	No

### Treatment

Our patients, all with complete ruptures, were treated surgically under general anesthesia. In all cases a standard deltopectoral approach was used, allowing for greater visualization of the rupture site. In 5/6 patients that rupture had occurred at the tendon’s insertion site, the insertion site on the humerus was identified, the tendon was mobilized and attached in the PM footprint at the lateral crista of the intertubercular groove, laterally of the long head of the biceps’ tendon, with suture anchors. In one patient with rupture at the musculotendinous junction an end-to-end suturing was performed. All surgical patients underwent the same physical therapist supervised rehabilitation regiment. Post-operatively, the arm was immobilized into 30° of abduction for 2 weeks. Passive and active range of motion exercises were initiated the 3^rd^ post-operative week.

### Outcomes

All ruptures occurred during bench press, without the presence of an assistant during training and with a mean weight of 183.4 kg for the three patients with the use of anabolic steroids and 147.6 kg for the patients without the invigoration of anabolic steroids. The average preinjury bench-press weight of 185.7 kg (range 170–205 kg) was restored to 160 kg (range 155–170 kg) postoperatively for the first group of anabolic steroids (86.1%) and from 152.5 kg (range 145–155) to 124 kg (range 115–130) (81.3%). Isokinetic strength evaluation revealed an average decrease of 9% (evaluation by an isokinetic dynamometer) without a statistically significant difference between the patients with or without the use of anabolic steroids. There was not any requirement for a second surgical procedure, and no postoperative wound complications or infections occurred.

The average follow-up time was 16 (range, 12–24) months. No complications were recorded. All patients were pain free. Full range of motion was obtained by the 6^th^ week after surgery. All operated patients returned to full activity in a mean 5.4 (range, 5–7) months postoperatively. The outcomes of the treatment were graded excellent, good, fair, or poor, as per the Bak criteria [3] (Table 2). All six patients who underwent surgical reconstruction of PT had excellent results with a full range of shoulder motion, almost normal muscle strength and fully restored anterior axillary fold (Fig. 1b).

**Table 2:** Bak criteria [3]

	Excellent	Good	Fair	Poor
Pain	Free	Free	With activity	Persistent
Range of Motion	Full	Slight decrease	Slight decrease	Restricted
Cosmesis	No complaints	Minor	Minor	Unsatisfactory
Return to Activity	Full function	Slight impairment	Impaired	Complications
Strength	< 10% isokinetic loss	< 20% isokinetic loss	> 20% isokinetic loss	> 20% isokinetic loss

All 3 patients who used anabolic steroids pre-operatively continued during the recovery period, as they stated during the follow-up period and one of them passed away within a year after his injury due to myocardial infarction.

## Discussion

Pectoralis major muscle is a broad muscle which lies on the anterior chest wall. It is a powerful adductor, internal rotator, and flexor of the humerus, that provides a dynamic stabilization of the shoulder joint. Although not essential for normal shoulder function, it is important in athletes and labor-intensive workers for producing maximal force in upper extremity movements. Furthermore, it forms the anterior axillary fold, which makes the muscle important for cosmetic reasons, especially to bodybuilders [5, 8, 10].

The PM covers a big part of the sternum, from the anterior surface of the sternal half to the attachment of the cartilage of the sixth or seventh rib [12]. Anatomically it consists of two heads; a clavicular head, arising from the medial half of the clavicle, and a sternocostal head, which comprises approximately 80% of the muscle. Fibers arising from the clavicle pass downwards and outwards, while those from the lower parts pass upwards and outwards. The fibers of the two heads converge like a fan that twist upon each other at 90^o^ before inserting on the lateral lip of the bicipital groove as a bilaminar tendon [8, 13, 14]. The medial and lateral pectoral nerves are responsible for the innervation of the PM muscle [12].

The overall incidence of PM rupture in the general population has not been estimated, but it has been reported to follow an increasing trend. This is more likely attributable to increased participation in weightlifting, body building and contact sports, in combination with increased use of anabolic steroids, increased awareness of this injury and the contemporary glorification of the male physique [4, 6, 15, 16]. Rupture of PM predominantly occurs in young males between 20 and 40 years of age [2, 5]. In our series the patients who did not use steroids were generally older (mean 4 years older) than the patients using steroids. Two of the three non-users were older than all the steroid users. This age difference could potentially explain the decreased weight threshold (about 40 kg less) for PM rupture.

The mechanism of injury mainly involves bench press weightlifting exercises, though less commonly injuries can result from forced extension or abduction against resistance or involuntary contraction [7]. With the arm positioned in extension of 30°, the short, inferior fibers of the sternocostal head undergo significant lengthening (30–40% of resting fiber length), and become susceptible to rupture under the application of high eternal loads [5, 6, 11]. In our series the non-dominant arm was affected in 50% of the cases approximately similar to the study of Marcin Kowalczuk et al. no difference between the dominant and non-dominant side was recorded (46,4% vs 49,1%) [17]. Οur working hypothesis was initially that greater strength and better neuromuscular coordination of the dominant limb would make it less vulnerable to pectoralis major tear. Αlthough it is necessary to study a larger pool of patients, it seems that there is probably a random pattern the weight slips to one side and the eccentric contraction of the pectoralis major, leading to rupture.

The broad use of anabolic steroids has been considered a risk factor for PM ruptures. Lifetime prevalence use for androgenic anabolic steroids has been estimated to be 3.3%, which is higher in males, 6.4% [18]. Athletes use anabolic steroids to build up muscular tissue and strength fast, and in many cases to improve their cosmetic physique. The long-term use of anabolic steroids has been related with serious adverse effects, including irreversible cardiovascular disease. The ultrastructural and biomechanical effects of anabolic steroids on tendons are poorly understood. Two alternative hypotheses have been suggested. One possibility is that high doses of anabolic steroids predispose tendons to rupture, by reducing their elasticity and rendering them stiffer; an increased stiff tendon absorbs less energy and fails with lesser elongation. A second possibility is that the anabolic steroids have little effect on tendons themselves, but they lead to increased muscle strength which becomes disproportionate to the strength of tendon [11, 19–21].

In our series, 50% of all patients had a confirmed history of anabolic steroids use prior to injury and after the repair. There has been an inconsistency in literature on the percentage of patients who sustained PM injury, while using anabolic steroids. In a study by Cordasco et al. all patients denied use of anabolic steroids, while Aärimaa and de Castro Pochini reported use of steroids in 36% to 90% of patients with PM rupture, respectively [6, 22, 23]. In a different study by de Castro Pochini et al. all included athletes who sustained PM rupture during weightlifting and half of the athletes who were injured while performing jiu-jitsu had a confirmed history of anabolic steroids use [24]. A recent systematic review of PM injuries associated the use of anabolic steroids with approximately 10% of the PM rupture cases. This inconsistency is more likely attributable to under-reporting of their use by the patients [6]. Ιt is important to mention that the athletes who used anabolic steroids in our study and suffered a torn PM lifted about 35 kg more than the bodybuilders who did not report the use of steroid substances. It is therefore possible that the cause that occasionally leads these athletes to rupture of the PM is not necessarily the pathophysiology of the muscle but the fact that the anabolic steroids lead to a rapid increase in muscle mass and therefore to an excessive upward adjustment of the athlete's goals in lifting weights.

Moreover, patients who used anabolic steroids before the injury continued without interruption during the postoperative recovery period. However, this fact had no negative effect either on the total recovery time or on the functional scores and the subjective criterion of satisfaction postoperatively in comparison with the disease control group of bodybuilders without the use of anabolic steroids.

The treatment options for the repair of PM injury involve both surgical and non-surgical modalities. The PM is not required for most activities of daily living, but it is necessary for strenuous activities of the upper arm. Additionally, injury of the PM can result in cosmetic deformity since it disrupts the anterior axillary fold. Thus, factors such as age, type of injury, activity level and cosmetic desires of the patient must be considered before deciding about management [8–11].

Surgical management is recommended for all complete tears of the PM, to restore full strength and function of the arm and to resume athletic activities in patients who require full use of the upper extremity, as well to restore cosmetic appearance of the muscle [8–11]. Several surgical techniques have been described in the literature, with the most described being trans-osseous tunnels, suture anchors and cortical buttons. Direct repair of the tendon stump, in case of tear at the musculotendinous junction and presence of sufficient healthy tissue attached to the insertion, has also been described with successful results [8–11]. All described techniques lead to excellent/good results in high percentages (over 80%) [25]. All six patients underwent surgical repair of PT and had excellent results with a full range of shoulder motion, almost normal muscle strength and fully restored anterior axillary fold. The average preinjury bench-press weight was restored postoperatively to 86.1% for the group of anabolic steroids users and to 81.3% for the other three patients. It is interesting to note that the isokinetic strength difference is only 9% compared to the functional bench-press weight. We therefore believe that the balanced strength and cooperation of the muscles at both arms is equally or even more important both for the maximum goal in weightlifting and for avoiding tears due to uneven load distribution.

The optimal timing for repair of PM rupture remains debatable. Acute reconstruction is performed within six weeks from injury and chronic after the 6^th^ post-injury week [2, 8]. Chronic ruptures can be more technically challenging and not amenable to direct repair. Antosh et al. reported sooner return to work and less pain with activity in acute-repair patients compared with the delayed-repair patients, though the differences were subtle [26]. Conversely, Schepsis et al. reported no significant, subjective or objective, difference between patients managed surgically for acute or chronic injuries [27]. Overall, delay in surgical treatment may result in a technically demanding procedure, but the clinical outcomes are comparable with acute repair and significantly better than non-operative treatment [8, 9]. In our series five patients underwent repair of avulsed tendons with suture anchors, in an acute and chronic setting respectively, and one patient with rupture at the musculotendinous junction underwent direct repair. Ιn agreement with the literature all patients regained full range of motion by the 6^th^ postoperative week and returned to full activity 5.6 months postoperatively regardless the chronicity of the repair.

## Conclusions

Rupture of the PM is an injury with increasing incidence within bodybuilding athletes due to the pronounced weightlifting programs during their training, which is probably not related to the dominance of the limb. The method of treatment is based on location, thickness, and chronicity of rupture. Fixation of the tendon with suture anchors results in excellent clinical outcomes and increased patient’s satisfaction postoperatively, regardless of the chronicity of the injury. Our observations in these cases suggest that anabolic steroids may contribute to the injury due to an excessive upward adjustment of the athlete's goals in lifting weights and moreover the continuation of administration even in the recovery period does not seem to have a negative effect either on the time or on the level of functional recovery postoperatively.

## Data Availability

The datasets used and/or analysed during the current study available from the corresponding author on reasonable request.

## Abbreviations

PM: Pectoralis Major
MRI: Magnetic Resonance Imaging
